# Editorial: Community series: towards a meaningful instrumental music education. Methods, perspectives, and challenges, volume II

**DOI:** 10.3389/fpsyg.2023.1303796

**Published:** 2023-11-02

**Authors:** Andrea Schiavio, Luc Nijs, Dylan van der Schyff, Marja-Leena Juntunen

**Affiliations:** ^1^School of Arts and Creative Technologies, University of York, York, United Kingdom; ^2^Department of Education and Social Work, University of Luxembourg, Esch-sur-Alzette, Luxembourg; ^3^Melbourne Conservatorium of Music, Faculty of Fine Arts and Music, University of Melbourne, Parkville, VIC, Australia; ^4^Faculty of Music Education, Folk Music and Jazz, Sibelius Academy, University of the Arts Helsinki, Helsinki, Finland

**Keywords:** instrumental music education, music, music education, music teacher, music student

Our Community Series introduces a comprehensive and dynamic perspective within the evolving field of instrumental music education. The first volume of this series, entitled *Towards a meaningful instrumental music education: methods, perspectives, and challenges*, examined a range of pivotal topics including technology, meaning, and expression. Likewise, the 11 contributions to this second volume are collaborative and draw upon a diverse spectrum of perspectives—they integrate empirical insights, engage robust theoretical frameworks, and develop reflective insights from pedagogical practice. In doing so, they enrich our comprehension of music's role within broader pedagogical contexts and showcase possibilities for catalyzing transformative shifts within music education itself. Like the first volume, this Research Topic provides insights that resonate with educators, researchers, and students alike.

The paper by Tullberg is an excellent example of a contribution that draws a strong link between theory and practice. This article addresses the concept of “affordances” in the context of engagement with musical instruments, highlighting a gap in the development of this concept within music research. The article emphasizes the importance of enhancing the application of the theory of affordances in the exploration, discussion, and development of innovative approaches to musical learning. It also underscores the ontological implications associated with the concept of affordances, urging caution when merging it with other theoretical domains or when incorporating it into empirical studies. Tullberg suggests that the analytical focus in studies involving musical instruments should be on the sensorimotor relationship that unfolds spatiotemporally during a musical event and concludes by discussing the educational implications stemming from this perspective as well as proposing avenues for further research in this field. Overall, Tullberg's work contributes to bridging the gap between theory and practice by elucidating the necessary components for a comprehensive understanding of the affordances of musical instruments.

Another important contribution based on a reflective integration of theoretical and practical insight is provided by Southcott and de Bruin. Their article employs a post-qualitative technique to delve into the intricate interplay between effective learning and personal experience in instrumental music education. Drawing from their own histories as instrumental performers and educators, they explore the connections between musicians, as learners and teachers, and their environments. The article discusses the experience of the authors, both of whom are performers (trumpet and clarinet, respectively) who also assume the roles of music educators. Through a continuous process of re-enactment and resistance against established norms, Southcott and de Bruin examine their transformative journeys from musicians to pedagogues. By delving into their multifaceted experiences, they strive to uncover insights into becoming the kind of teacher they aspire to be. This pathway is extended to their students as they explore the question of how one can evolve into a better music educator. This journey, according to the authors, is a perpetual series of turns, each leading to new avenues of being and becoming.

Morgan and O'Neill provide an empirical study that employs a mixed-methods approach to explore the relevance of European classical music within the context of music education in the United Kingdom. Notably, the study sheds light on the enduring pedagogical value of J.S. Bach's compositions, contributing to the broader conversation about curriculum diversification and the preservation of historically significant musical contributions. The authors conducted semi-structured interviews with music educators and implemented a listening experiment. The experiment involved participants rating their emotional responses to pieces composed by Bach, Beethoven, and Mozart. A reflexive thematic analysis was employed to defend the inclusion of Bach's music in mainstream education. Additionally, participants' emotional responses were quantified using a standardized scale, while ratings for valence, arousal, familiarity, and overall enjoyment were also collected. The outcomes reveal a statistically significant connection between the music of specific composers and certain emotion categories, suggesting that the emotional impact of music can vary depending on the composer. Such findings, it is argued, may potentially confirm the continued admiration for J. S. Bach in music education.

Another important empirical contribution in our Research Topic is by Lippolis et al.. Their study explores the impact of musical learning on behavior and cognitive development during the transition from childhood to adulthood. Amid the multitude of music training programs, the study zeroes in on instrumental training integrated into the public middle school curriculum in Italy. This involves a comprehensive curriculum encompassing individual, group, and collective (orchestral) lessons multiple times a week. Conducted at three middle schools, the research tested 285 preadolescent children aged 10–14 years. The test and questionnaire battery included adaptive assessments for visuo-spatial working memory skills, fluid intelligence, and music-related perceptual and memory abilities. The study found significant distinctions between students in the music and standard curricula across both perceptual and cognitive domains. These differences persisted even when accounting for pre-existing individual variations in musical sophistication. Among the noteworthy findings, preadolescents enrolled in the music curriculum during their third and final year of middle school exhibited superior performance and the most substantial advantage compared to the control group in both audiovisual working memory and fluid intelligence. Taken together, these findings—although not establishing causality—provide valuable insights that can steer future research toward longitudinal assessments, contributing to the ongoing dialogue about the integration of music training and cognitive development during this pivotal life stage.

Kruse-Weber et al. present a comprehensive case study centered on a professional development project aimed at facilitating collaborative reflection among instrumental music teachers. This qualitative study underscores the vital role of collaborative reflection in professional and social growth. The investigation, conducted by a university research team in collaboration with 13 music educators from a Styrian music school in Austria, spanned from 2019 to 2021, including the initial phase of the COVID-19 pandemic. The study addresses several research areas: the participants' perspectives on collaborative professional development, their use of reflection tools, identification with workshop interventions, factors impacting the outcomes of reflection tools, their evolving thinking and attitudes, group dynamics, and the development of trust among participants. Thematic analysis of the data generated five themes: forming group cohesion, inspiring and appreciating collaboration, bridging theory and practice, addressing challenges and potentials during the pandemic, and identifying the music school's identity and significance. This research shows how a collaborative reflective approach not only enriches professional development but also contributes to redefining the identity of music schools and teaching practices.

The qualitative study by Stolp et al. brings together theory and practical insights, this time bridging the theoretical concepts of agency and entrainment within the realm of music education. Their paper involves an interview-based approach that delves into the interplay between these two concepts in collaborative music-making, as reported by students and teachers. Drawing from the experiences of 23 Grade 6 and 11 students and their music teachers from various primary schools, the study investigates how agency and entrainment interact to shape joint musical activities. The authors identify four central themes: presence, belonging, safety, and continuity. These themes encapsulate the nuanced relationship between agency and entrainment in the context of classroom-based joint music-making. The study's findings offer insights into the complementary experiences of students and teachers during collaborative musical endeavors. This exploration provides educators with a unique perspective on the potential of joint music-making to foster group cohesion and social interaction, rendering it a platform for the cultivation of agency among participants.

Looking directly at more contextual pedagogical settings, the contribution by Jordhus-Lier et al. examines music teachers' content-related decision-making processes in Norwegian municipal schools of music and arts. These publicly funded institutions offer extra-curricular activities in music and other art forms for children and adolescents. The study recognizes that teaching content is central to whether students feel included or excluded, indicating that music teachers' beliefs and actions play a pivotal role in shaping the learning environment. To uncover the approaches that teachers take to select content and methods in instrumental music teaching, the authors conducted a survey among 151 music teachers and interviewed 11 music teachers. The findings reveal several meaningful approaches that guide music teachers' decision-making processes. These approaches emphasize the centrality of students in content selection, advocating for genre versatility that exposes students to a broad range of musical genres and styles.

Schiavio and Nijs' contribution also explores practical and pedagogical contexts. The authors devised a novel collaborative online music course, emphasizing creativity, interaction and bodily movement. In this course, four musical novices learned how to play the clarinet. Despite the challenges inherent in distance learning environments, the study's goal was to explore the learning experience and outcomes of the participants. To do so, the authors conducted semi-structured interviews with the learners and employed thematic analysis to gain insights into their experiences. The interviews revealed several key themes. Firstly, the establishment of meaningful relationships with the musical instrument was identified as crucial for building musicality. The participants highlighted how their connection with the clarinet deepened over time, enabling them to engage more effectively with the instrument and the music they produced and contributing to a more enriching learning experience. Secondly, building relationships with fellow students was highlighted as important. Collaborative learning allowed the novices to share their experiences, challenges, and successes with each other. This interaction fostered a sense of community and camaraderie, promoting a supportive and motivating environment for learning. Furthermore, the interplay between creativity and control emerged as a notable aspect of the learners' experiences. The study showcased how the learners were encouraged to engage in creative and expressive music-making activities, which enhanced their understanding of musical concepts and techniques. The study suggests that remote music tuition within a small group can serve as a valuable resource for individuals starting their musical training. Additionally, it emphasizes the positive impact of a teaching approach that integrates creativity, collaboration, and bodily movement. This approach not only addresses the challenges of distance learning but also suggests possibilities for promoting musical growth and wellbeing in an online pedagogical context.

The remaining contributions cover three fundamental aspects that, in one way or another, encompassing many of the previous studies: technology, meaning, and expression.

The study conducted by Michałko et al. contributes to the exploration of interactive technologies in instrumental music education. As current research and theory increasingly recognizes the potential of interactive tools to support learning and teaching methods, the gap between proposed technological solutions and their integration into daily teaching routines remains evident. The authors conducted an online survey involving violin and drum kit teachers to address this gap. Their findings unveil distinct learning profiles among novice violin and drum kit students, reflecting the diverse teaching approaches employed for adults and children. This differentiation underscores the need for adaptable and tailored teaching methods based on the age and background of the learners. Moreover, the study delves into teachers' perspectives on the use of virtual reality and smart wearable technologies for early instrumental training. Teachers' opinions and attitudes toward technology design are highlighted, emphasizing the importance of involving educators in the initial stages of technology development. This approach ensures that the technology aligns with the practical requirements of teaching and effectively addresses the actual needs of both teachers and students.

The article by Tullberg and Sæther delves into the concept of meaning within the context of musical learning, focusing on instrumental music education. The authors emphasize the role of social interactions in shaping a meaningful music education, using Swedish folk music as a backdrop for their exploration. Their objective is to provide analytical tools that shed light on the intricate processes through which meaning is negotiated within the context of learning music. To achieve this, the authors employ a theoretical foundation based on situated cognition, situated learning, and communities of practice. Central to their analysis is the concept of “negotiations of meaning”. They view these negotiations as continuous, evolving processes that offer insights into how individual learning experiences, educational contexts, and the broader musical environment intersect and influence each other. The authors offer practical application of their analytical framework through an ongoing research project centered on various communities within Swedish folk music. They showcase how specific aspects such as the identity of a musician and approaches to notation serve as pivotal points for meaning negotiations across diverse communities. The authors adopt an ethnographic approach, embracing the concept of “messy research” and incorporating musical research sensibilities and stepwise-deductive induction. This methodological mix allows for a comprehensive exploration of the intricate dimensions involved in negotiating meaning within musical learning environments. The article contributes to a deeper comprehension of how meaning takes shape and evolves in the realm of instrumental music tuition, thereby offering valuable insights into the pedagogical dynamics that shape music learning experiences.

Finally, the paper by Juntunen et al. presents a unique theoretical framework rooted in Merleau-Ponty's phenomenological philosophy to explore the concept of expression in popular music singing and its implications for pedagogy. Departing from the conventional understanding of expression, the study delves into an embodied perspective, focusing on the interconnectedness of intentionality, body schema, gesture, reversibility, and intersubjectivity to illuminate the intricate and multifaceted nature of vocal expression in singing. The authors advocate for the conception of expression as an intentional endeavor, driven by the holistic functioning of the body, and guided by the content of the song's lyrics and intended emotions. They use the notion of a “free voice” to characterize healthy vocal production. It enables the immediate manifestation of expression through voice and gestures that give life to the intended meaning. In emphasizing the interpersonal aspect, the article highlights the interactive and intersubjective process through which performers and listeners mutually influence each other. The concept of reversibility, which intertwines perception and the perceived object, underscores the inseparability of action and perception, while acknowledging the perpetual gap in fully comprehending one's own expression. Amidst their theoretical discourse, the authors offer pedagogical insights that align with their phenomenological perspective. They propose a shift away from primarily technical approaches to expression in singing, advocating for a holistic integration of body understanding and trust in voice production and expression. The authors also suggest that the teaching of expression should not be separated from the teaching of vocal technique, but integrated into it. By viewing expression as an internal process rooted in one's personality and emotional experiences, they advocate for an inside-out approach to nurture authentic and meaningful vocal expression.

In all, the contributions in our Community Series enhance understanding of instrumental music education. They combine diverse viewpoints, empirical research, and pedagogical insights, fostering a transformative impact. This synthesis empowers educators, students, and researchers and provides tools for enriching learning environments. The volume encourages reflective engagement and evidence-based practices in music pedagogy. It promotes continuous growth and adaptation, leading to improved teaching methods, inclusive learning environments, and lifelong learners engaged with complexity and challenges of music and its praxis.

## Author contributions

AS: Writing—original draft, Writing—review & editing. LN: Writing—review & editing. DS: Writing—review & editing. M-LJ: Writing—review & editing.

